# Impact of Adult *Popillia japonica* (Coleoptera: Scarabaeidae) Foliar Feeding Injury on Fruit Yield and Quality of a Temperate, Cold-Hardy Wine Grape, ‘Frontenac'

**DOI:** 10.3389/finsc.2022.887659

**Published:** 2022-04-26

**Authors:** Dominique N. Ebbenga, Eric C. Burkness, Matthew D. Clark, William D. Hutchison

**Affiliations:** ^1^Department of Entomology, University of Minnesota, St. Paul, MN, United States; ^2^Department of Horticultural Science, University of Minnesota, St. Paul, MN, United States

**Keywords:** invasive species, defoliation, soluble solid content, titratable acidity, artificial infestation

## Abstract

*Popillia japonica* (Newman), is a highly polyphagous, invasive species, first recorded in the U.S. in 1916, and detected in Minnesota in the late 1960s. Historically, research on this pest in the Midwest U.S. has focused primarily on ornamental and turf crops, with little attention placed on adult feeding damage to fruit crops. Recently, wine grape producers in the region noted substantial increases in defoliation from *P. japonica* feeding, confirming concerns for this perennial high value crop. To address these concerns, studies were conducted during the summers of 2020–2021 to understand the impact of *P. japonica* foliar feeding on the quality and yield of wine grapes. Trials utilized vines of the wine grape variety, ‘Frontenac.' In addition to open plots, whole vines were caged within fine mesh netting and infested with *P. japonica* at 0, 25, 50, and 100 beetles per meter-row of vine. Beetles used for infestations were collected from natural field populations of *P. japonica* and left to feed until grapes were ready for harvest. During harvest, data collection included leaf samples for obtaining average percent defoliation, cluster weights, and berry subsamples for soluble solid content, pH, titratable acidity, and phenolic compound measurements. Results from these studies demonstrated that as beetle population density and defoliation per m-row increases, at-harvest measurements of quality parameters are significantly and negatively affected (*P* < 0.05) when compared with uninfested vines. The negative impacts to fruit quality exhibited in these studies will be important in the development of future management strategies for *P. japonica* in ‘Frontenac.'

## Introduction

Japanese beetle, *Popillia japonica* Newman (Coleoptera: Scarabaeidae), is an invasive insect, native to Japan (1). The species was first detected in the United States in New Jersey in 1916, and eventually found in Minnesota in 1968 (2). Since the arrival in the U.S., *P. japonica* have become a major pest in turfgrass, ornamental and horticultural settings (3, 4). In response to the beetle's pest status in the U.S., a quarantine program has been in place for most western states to prevent further spread and establishment (5, 6). *Popillia japonica* exhibit a holometabolous life cycle. The larvae, or white grubs, live beneath the soil surface feeding on the roots of various grasses, potentially killing turfgrass and causing aesthetic damage to lawns and golf courses (7). Conversely, the adult life stage lives above ground and feeds on the fruit, flower, and foliage of more than 300 different plant species; primarily focusing on the foliage by defoliating the leaves in a characteristic pattern often referred to as skeletonization (3, 8).

Most of the published research on *P. japonica* has been focused on the biology, phenology, and control of the immature larval instars, particularly to lawns and golf courses (7, 9), while relatively little research has explored the impact of *P. japonica* adult feeding on horticultural crops (4, 5). When considering wine grapes (*Vitis vinifera* L. and *Vitis* hybrids), a highly preferred host of adult *P. japonica*, only a few studies have examined the impact of high defoliation rates on yield or juice quality (10–13). Because wine grapes depend on adequate leaf surface area for photosynthesis to provide sugars and non-structural carbohydrates vital to survival and fruit development (14), it is critical to understand the impacts of defoliation. As described by Pfeiffer (14), early season leaves are soft and delicate and photosynthesize sunlight to encourage shoot and cluster growth. Later in the season, during véraison (i.e., when fruit begins to change color), leaves become more tough and can tolerate more leaf feeding, and individual berries begin to become the main sink for sugars accumulated through photosynthesis (15). Understanding the susceptibility of different growth stages can be important when looking at *P. japonica* phenology in Minnesota. *Popillia japonica* adults typically emerge in late June to early July and populations persist until mid-October (16). This period of adult activity overlaps with most of the wine grape growth stages including when leaves are delicate, véraison, and through harvest. Because the foliar feeding activity of adult *P. japonica* and susceptible growth stages of wine grapes coincide, wine grapes in Minnesota may be at high risk for negative impacts to grape berry yield and quality.

Fruit quality refers to juice attributes of grapes both prior to and at harvest that determine the eventual quality of wine contributing to organoleptic properties, storage, stability, and color (15). Examples of this would be soluble solid content (SSC in °Brix) which estimates roughly as the percent sugar in a grape berry and depending on the value, can be a predictor of the percent alcohol in the subsequent wine and impacts flavor. SSC is typically measured prior to harvest in the field to inform growers when harvest should occur in addition to tasting and visual cues (17). Another quality parameter is total titratable acidity (TA), which measures the acid content in the grape juice and will impact the flavor of the subsequent wine. TA is typically measured after the grapes have been harvested (18) and if needed, wine-making practices can be used to adjust the wine flavor. A few other parameters include pH which will further describe the acidity, and finally phenolic compounds found in the grape skin and measured after grapes have been crushed to determine the color and sensory properties (tannin content) of the wine (19). Depending upon the region and the variety of grapes grown, different harvest recommendations are made to ensure the best quality wine possible (19). Furthermore, yield refers to the weight of clusters at the time of harvest.

In Minnesota, wine grapes account for over $80 million dollars in economic activity making it a high value crop that growers and other stakeholders wish to protect (20). The paucity of data for how defoliation by *P. japonica* affects wine grapes, both within season and over multiple growing seasons of a perennial crop, leads to many concerns for how to proceed agronomically and the financial impact that may occur. Current pest management strategies for *P. japonica* adults, in Minnesota wine grapes, rely heavily on the use of insecticides (3). However, there are currently no research-based action or economic thresholds for this pest in vineyards. If *P. japonica* adults are present at any level in the crop, insecticides may be applied for management. Furthermore, in times of high population density, insecticides can be sprayed as often as weekly intervals (personal observations). These excessive insecticide applications may cause unnecessary environmental impacts and gives cause to continue research on this pest's impact to wine grapes.

We therefore developed the following research objectives to better understand the degree to which *P. japonica* feeding activity may affect wine grape yield and juice quality under a midwestern U.S. climate, to better guide the development of integrated pest management (IPM) strategies. Our study focused specifically on a popular cold-hardy Minnesota wine grape variety, ‘Frontenac' (21), to determine potential yield impacts, as well as fruit and juice quality, in response to a range of *P. japonica* infestation levels. However, results from these studies could also be beneficial and applicable to other regions *P. Japonica* has invaded.

## Materials and Methods

### Impacts on Fruit Yield and Quality

**Figure 1:**
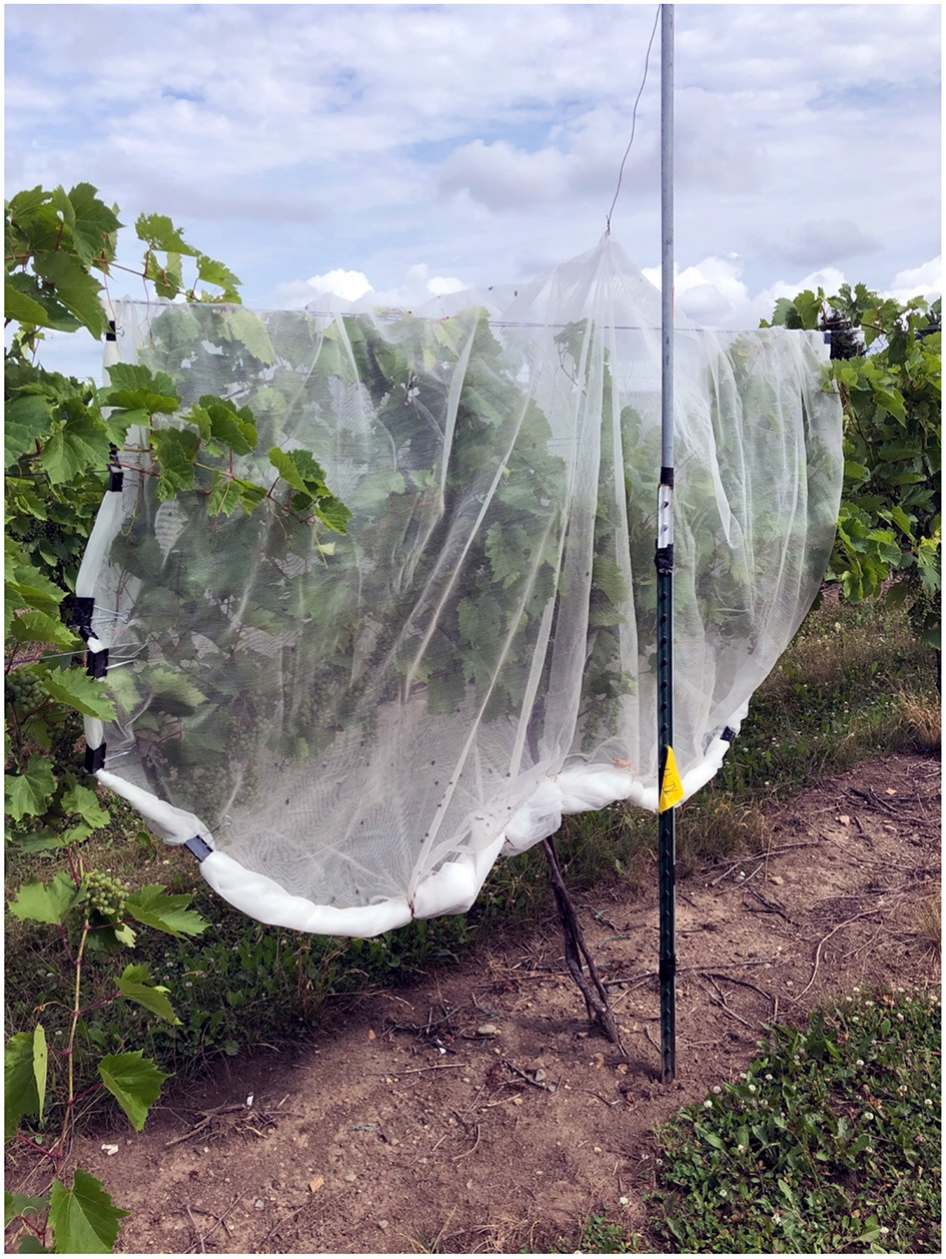
Example of exclusion netting plot used in trials. Netting was tossed over the top wire, rolled up on all edges and fastened with binder clips and wires to seal the netting. Poles were installed with wires attached to elevate the netting above the canopy.

Trials to assess the impact of *P. japonica* feeding and subsequent defoliation on fruit yield and quality were conducted at a 25-hectare production vineyard near Prior Lake, Minnesota (44.621776N, −93.442489W) in 2020 and 2021. Vines used in experimental plots were 6 or 7 years old depending on the year studies were conducted. Experimental plots were established on 19 June 2020, and 21 June 2021, during the wine grape growth stage known as ‘pea-size berry' (22). This timing for plot establishment was selected because it occurs after flower pollination, so that netting would not interfere with fruit set, but before *P. japonica* emergence (16), to minimize any foliar injury from natural beetle populations prior to infestations of the netted vines. A total of 5 treatments were evaluated each year and consisted of: (1) an open plot to allow for natural beetle feeding, (2) a netted check plot with zero-beetle, (3) a netted plot infested with 25 beetles/m-row, (4) a netted plot infested with 50 beetles/m-row, and (5) a netted plot infested with 100 beetles/m-row. Each treatment was replicated 4 times in a randomized complete block design, within one trellised row measuring ~156 m, of ‘Frontenac' wine grapes selected each year. Netted plots each used a 3 m by 4 m piece of 80-gram mesh netting (ExcludeNet, Tek-knit Industries, Quebec, CA) to cage an entire vine (23, 24) measuring to be 2 m in length. A previous study, under Minnesota growing conditions, showed that the same 80-gr mesh netting product did not significantly affect ambient temperatures during summer, compared to open plots (23). To keep the netting from resting on top of the plant canopy, 3.96 m poles were installed with wires attached that maintained the netting elevated above the canopy. To secure the netting around the vines, the ends of the netting were rolled into each other and then held together using large and medium steel binder clips (Office Depot Inc., Boca Raton, FL). In areas where clips did not create a tight enough closure around vines and trellis wires, 15- gauge wire was used to tightly wrap and secure netting (24). An example of a netted plot used in trials can be seen in Figure 1.

Infestations of treatments occurred on 15 and 9 July in 2020 and 2021, respectively, during the ‘pea-size berry' growth stage. Beetles for infestations were collected from naturally occurring populations in a raspberry research field at UMore Park near Rosemount, MN (44.727839N, −93.097000W), placed in ventilated containers and transported immediately to the vineyard in Prior Lake for same-day release in the caged plots. Once infestation occurred, the beetles were allowed to feed on vines until harvest. After infestation, vines were observed weekly to assess the health and feeding activity of the beetles, ensure cages were securely sealed, and to take beetle counts in 1 m of row of each open plot.

Harvest of leaf, cluster and berry samples from experimental plots occurred on 5 Oct. 2020, and earlier on 9 Sept. 2021, due to drought conditions causing concern for loss of leaf samples; methodology was similar to that of Ebbenga et al. (25). For each plot, 10 randomly selected leaves were collected and placed in 20 cm by 25 cm re-closable bags (Minigrip Re-closable bags, Consolidated plastics, Stow, OH) to obtain average percent defoliation for each treatment using the LeafByte app (26). Additionally, all clusters were hand harvested from each plot and placed into 49 L plastic bags (Warp Bros., Chicago, IL) and weighed on a Doran 8000 digital scale (Doran Scales Inc. Batavia, IL) to obtain the 1-m row average weight. After weights were obtained, 5–7 clusters were randomly selected from the harvested clusters for each plot. Among these clusters, 10 randomly selected berries were removed from the remaining clusters from each plot and placed in individual 30-ml cups (Dart Container Corp., Mason, MI) and capped. The selected clusters and samples of berries were placed in coolers and immediately transported to the University of Minnesota's Horticultural Research Center, Grape Breeding and Enology Laboratory in Excelsior, MN for processing.

### Laboratory Processing for Quality Parameters

Once the 5–7 clusters of grapes arrived at the enology lab, the same day as harvest, they were juiced, utilizing methods similar to Ebbenga et al. (25). The juicing process consisted of placing the clusters from each plot in a 1-gallon Ziploc^®^ bag (S.C. Johnson & Son, inc., Racine, WI), and crushed by hand. Once crushed, the subsequent juice was poured and pressed through a stainless-steel China Cap Strainer (New Star Foodservice, Chino, CA). The resulting juice was collected in a Falcon 50 ml Conical Centrifuge Tube (Fisher Scientific, Hampton, NH) and used to measure pH, and titratable acidity (25). In 2021, a second vial of juice was collected, labeled, and stored in a freezer at −62°C until the samples were delivered via overnight express shipment to the ETS laboratories (St. Helena, CA) to obtain measurements of tannin, polymeric anthocyanins, total anthocyanins, and polymeric anthocyanins/tannin index. Lastly, the 10 randomly selected individual berries placed in individual 30 ml plastic cups with lids (Dart Container Corp., Mason, MI) from each plot were used to obtain average SSC using a refractometer (Shen Zhen YIERYI Technology Co., Ltd Shen Zhen City, Guang Dong Province, China) (27).

### Statistical Analysis

Data were analyzed using analysis of variance (ANOVA) with R statistical software (28). Trials were established in a randomized complete block design (RCBD). To achieve this, replications were made into four blocks and each of the five treatments were randomized within each block. Analysis comparing the treatments of the different beetle densities and replications were included in the linear model. When significant differences were found, a mean separation was conducted using Tukey's honest significant difference test [Agricolae, HSD.test, (29)]. Analytical assumptions for a one-way ANOVA were met prior to analysis and no transformations were conducted on the data.

## Results

**Figure 2:**
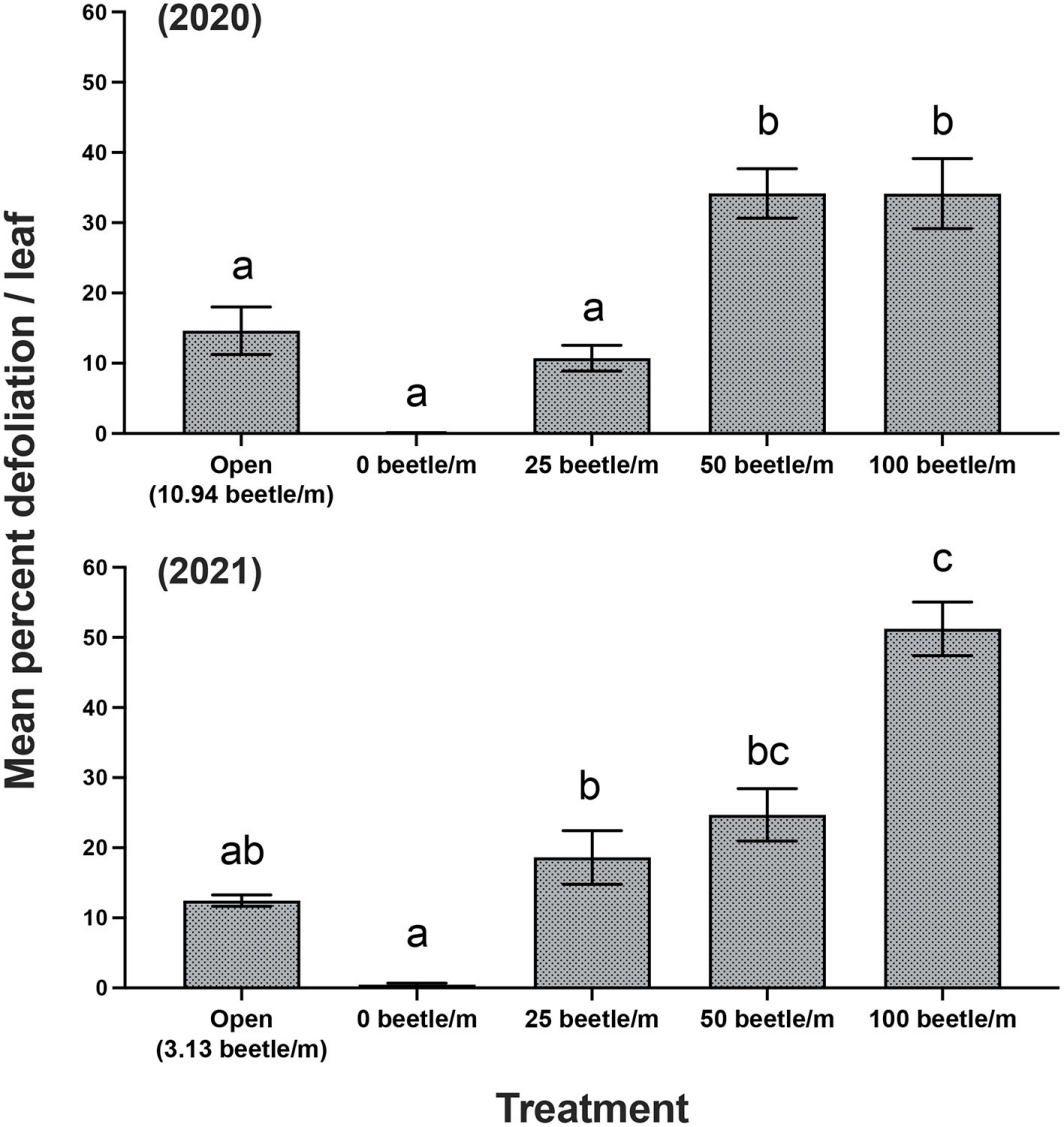
Mean (±SEM) measurements of mean percent defoliation per leaf for years 2020 and 2021. Bars within a year followed by the same letter are not significantly different (*P* > 0.05); ANOVA with Tukey's HSD mean separation test.

**Figure 3:**
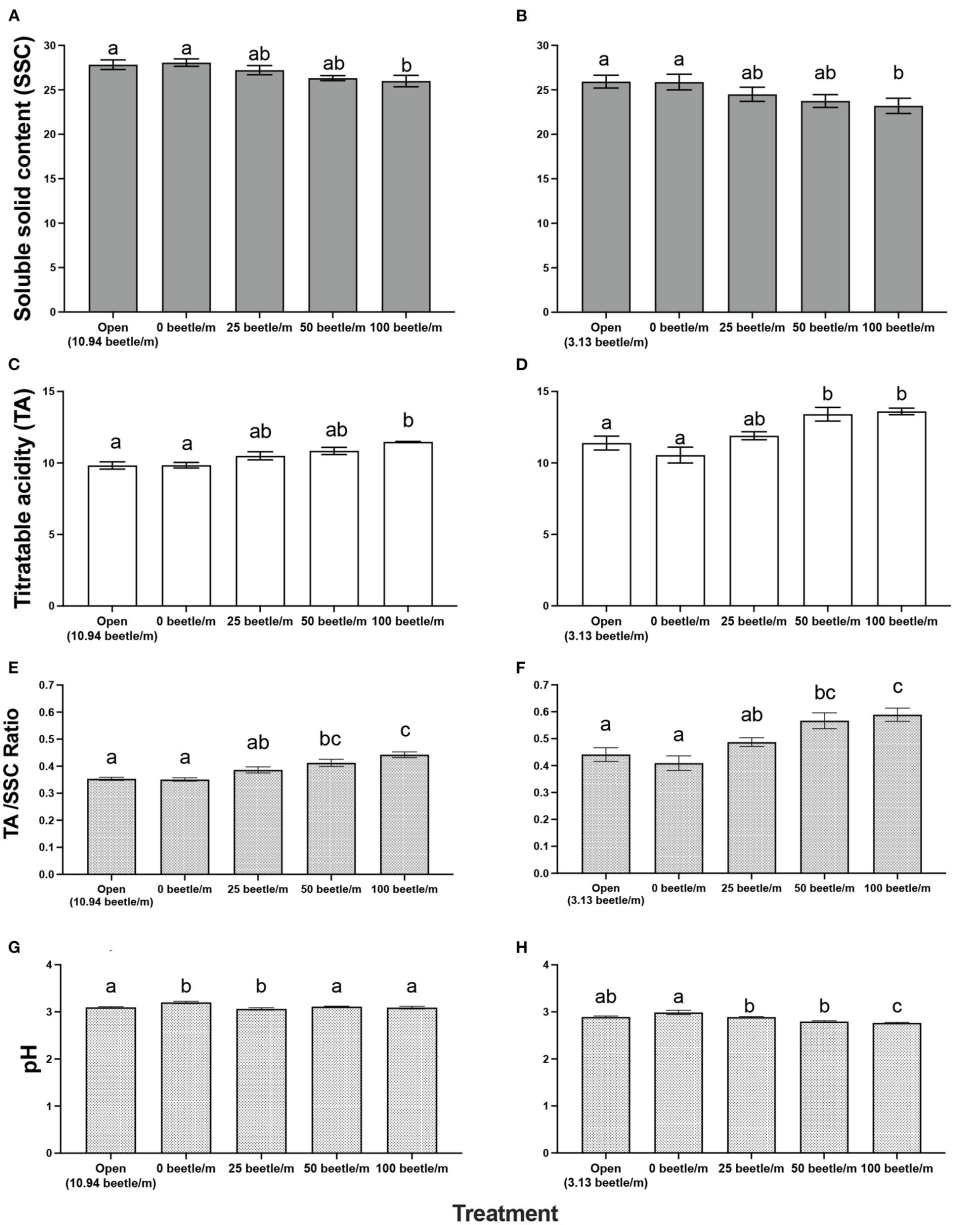
Mean (±SEM) measurements of fruit quality for a 10-berry subsample for soluble solid content (SSC in °Brix) in 2020 **(A)** and 2021 **(B)**, titratable acidity in 2020 **(C)** and 2021 **(D)**, Acid/SSC Ratio in 2020 **(E)** and 2021 **(F)**, and pH in 2020 **(G)** and 2021 **(H)**. Bars within a year followed by the same letter are not significantly different (*P* > 0.05); ANOVA with Tukey's HSD mean separation test.

For 2020, open plots averaged 10.94 *P. japonica* per m-row, observations of zero-beetle per m-row plots remained at zero, and all other treatments had the sufficient amount of beetles present in the cages based on infestations. Mean percent defoliation across plots based on leaf samples collected at harvest exhibited a significant increase [*F*_(4, 12)_ = 19.050, *P* < 0.001] as beetle densities increased with the highest beetle density of 100 per m- row showing approximately 35% defoliation; this compared to near 0% defoliation with the zero-beetle per m-row treatment (Figure 2). Results from the fruit quality analysis indicated that several parameters were significantly affected by *P. japonica* defoliation. For the SSC results, we observed a significant decrease [*F*_(4, 12)_ = 5.514, *P* = 0.009] in accumulation of SSC in grape samples as beetle densities and subsequent defoliation increased (Figure 3A). The TA parameter demonstrated a significant increase [*F*_(4, 12)_ = 8.4118, *P* = 0.002] in acidity levels in treatments when higher densities of beetles and defoliation occurred (Figure 3C). Furthermore, SSC and TA parameters from these trials indicate that at about 50 beetles per m-row, and >30–35% defoliation we begin seeing undesirable trends where SSC values are lower and TA values are greater, relative to our treatments with little to no beetle feeding occurring. To further demonstrate the differences across treatments, a ratio of TA to SSC was also examined. Data from this analysis affirms that as treatments increase in beetle density and subsequent defoliation, there is a significant increase [*F*_(4, 12)_ = 19.938, *P* < 0.001] in the TA/SSC ratio (Figure 3E). The pH results obtained for 2020 indicated a significant and negative trend in pH, or higher acidity [*F*_(4, 12)_ = 7.042, *P* = 0.004], as percent defoliation increased (Figure 3G). In contrast to the juice quality parameters, however, yield in 2020, measured based on the mean weight of clusters per m-row, did not show significant differences [*F*_(4, 12)_ = 0.8650, *P* = 0.5124] in response to infestation treatments. Mean (±SEM) weight (kg) for yield was 1.59(±0.01) kg/m-row for the open plot treatment, while the netted plots yielded 1.71 (±0.28), 1.61 (±0.26), 1.59 (±0.28), and 2.14 (±0.35) kg/m-row, for 0, 25, 50 and 100 beetles per m-row, respectively.

For 2021, the open plots averaged 3.13. *P. japonica* per m-row, while observations for the zero-beetle per m-row plots remained at zero, and all other treatments had the sufficient amount of beetles present in the cages based on infestations. Similar to results in 2020, leaf samples used to obtain average percent defoliation across plots showed significant differences [*F*_(4, 12)_ = 44.977, *P* < 0.001]; as beetle densities increased with the highest beetle density at 100 per m- row showing 51% defoliation, compared to near 0% defoliation with the zero-beetle per m-row treatment (Figure 2). Results for fruit quality parameters again demonstrated significant differences in SSC [*F*_(4, 12)_ = 5.861, *P* = 0.009] in accumulation of SSC in grape samples as beetle densities and subsequent defoliation increased (Figure 3B). Next, our TA parameters demonstrated a significant increase [*F*_(4, 12)_ = 8.649, *P* = 0.002] in acidity levels as treatments increased to higher densities of beetles and defoliation (Figure 3D). For a second year, our SSC and TA parameters trials indicate that at about 50 beetles per m-row, and >30–35% defoliation we begin observing the negative impacts of decreased SSC and increased TA values. Ratios of TA to SSC data demonstrate again that as treatments increase in beetle density and subsequent defoliation, there is a significant increase [*F*_(4, 12)_ = 12.287, *P* < 0.001] in the TA/SSC ratio (Figure 3F). Results obtained from our pH in 2021 observed a significant [*F*_(4, 12)_ = 10.815, *P* < 0.001] and negative trend for pH values as percent defoliation increased (Figure 3H). Similar to 2020, there were no significant differences in yield [*F*_(4, 12)_ = 0.593, *P* = 0.674] across infestation treatments. Mean (±SEM) weight (kg) for yield was 4.96 (±0.53) kg/m-row for the open plot treatment, while the netted plots yielded 5.44 (±0.88), 5.05 (±0.32), 4.48 (±0.58), and 4.43 (±0.13) kg/m-row, for 0, 25, 50 and 100 beetles per m-row, respectively.

Also in 2021, juice samples analyzed for measurements of phenolic compounds demonstrated additional negative impacts by *P. japonica*. For example, significantly lower polymeric anthocyanins [*F*_(4, 12)_ = 5.516, *P* = 0.009], total anthocyanins [*F*_(4, 12)_ = 6.608, *P* = 0.005], and polymeric anthocyanins/tannin index [*F*_(4, 12)_ = 4.882, *p* = 0.014] were observed as beetle density and subsequent defoliation increased (Table 1). Phenolic compound measurements for the tannin index alone did not show significant differences across treatments [*F*_(4, 12)_ = 2.883, *P* = 0.069]; however, we observed a decreasing trend in tannin values as beetle density increased.

**Table 1:** Measures of juice quality parameters for the wine grape variety Frontenac, under different infestations with *P. japonica*, Prior Lake, MN, 2021.

**Treatment**	**Tannin (mg/L)**	**Polymeric Anthocyanins (mg/L)**	**Total Anthocyanins (mg/L)**	**Polymeric anthocyanins/tannin index**
	**Mean (±SEM)**	**Mean (±SEM)**	**Mean (±SEM)**	**Mean (±SEM)**
Open (3.13 beetle/m)	86.25 (±3.97) a	4.00 (±0.41) b	860.25 (±94.11) ab	0.046 (±0.003) b
Net 0 beetle/m	92.00 (±3.32) a	5.25 (±0.25) a	1073.50 (±136.74) a	0.057 (±0.001) a
Net 25 beetle/m	84.25 (±2.50) a	4.00 (±0.00) b	776.25 (±28.11) ab	0.048 (±0.001) ab
Net 50 beetle/m	81.25 (±1.98) a	4.00 (±0.00) b	559.75 (±49.14) b	0.049 (0±.001) ab
Net 100 beetle/m	81.75 (±1.11) a	3.74 (±0.25) b	516.50 (±61.62) b	0.046 (±0.003) b

^a, b^*Means within columns followed by the same letter are not significantly different (p > 0.05); ANOVA with Tukey's HSD mean separation test. Results from ETS laboratory analysis, juice samples collected on Sept. 9, 2021*.

## Discussion

In this study, we conceived research objectives to better understand the degree to which *P. japonica* feeding activity may have a negative impact on fruit yield and quality of wine grape, ‘Frontenac,' and thus provide results that would be beneficial to growers who are motivated to improve their IPM programs. Overall, our results suggest that yield was not significantly impacted by an increase in *P. japonica* density. Any differences observed for yield in our study was more likely due to the natural variation in vine production or abiotic stressors for that given year, vs. the impacts of *P. japonica* treatments. However, data collected on the fruit quality parameters produced numerous significant impacts as beetle densities and subsequent defoliation increased.

Fruit quality parameters of ‘Frontenac' at harvest demonstrated negative impacts to the SSC (°Brix) values among the different treatment levels of beetle infestations (Figures 3A,B). In a wine grape variety such as ‘Frontenac,' grapes should measure between 22 and 25 °Brix and could be as high as 30 °Brix for dessert wines per recommendations from the University of Minnesota (30). While SSC for every treatment falls within this recommended harvest threshold, the data demonstrate that *P. japonica* feeding is impacting the plants' ability to properly photosynthesize and accumulate sugars in berries in an efficient manner (14). This becomes especially concerning in a cold climate such as Minnesota, because delaying harvest to boost SSC could place a grower at risk of losing the crop to an early frost, or berry breakdown for some varieties in some years.

For both years, treatments applied to TA resulted in a significant increase in acidity levels as beetle densities increased (Figures 3C,D). High TA in the grape juice can have an undesired impact on the flavor of the wine as it relates to the acid/sugar balance in the finished wine product. In a cold hardy wine grape variety such as ‘Frontenac' with naturally high acidity, it is vital the acidity level remain below 15 g/L as described by Clark (30). Per these recommendations, we can note all our treatments remain below this threshold having little impact on the subsequent wine. However, it is still important to know that even though values remain within the recommended threshold, we again observe the negative impacts *P. japonica* feeding has on the wine grape berry quality.

Values for pH in both years also demonstrate significant differences across treatments (Figures 3G,H). Current recommendations for pH are set to a value between 3 and 3.3 (30). In 2020, harvest of our trials occurred during the typical harvest window for ‘Frontenac' in Minnesota. All pH values in 2020 were within the recommended threshold, but we still observed a negative trend for pH values as percent defoliation increased. In 2021, due to drought conditions during the season, harvest occurred sooner than recommended as to avoid loss of defoliated leaf samples. However, this does not negate the results collected from this trial. While we cannot comment on the pH being within recommended parameters, we see a similar pattern of negative trends as percent defoliation of the crop exceeded 30%, and >50 beetles per m-row were placed in a cage.

Finally, grape juice samples in 2021 that were analyzed to capture the phenolic compounds demonstrated significant differences in some measurements when compared to the zero-beetle treatment (Table 1). Like SSC, TA, and pH, these results alone do not make the wine unmarketable, but the impacts made by *P. japonica* feeding add an extra layer of work and consideration in the wine making process. For example, looking specifically at the tannin measurements, while it may not be statistically significant, we see a numerical trend for a decrease in tannin with the increase in beetle density treatments. Tannins not only impact the flavor and mouthfeel of the wine, but they also help wine retain its color, also referred to as anthocyanins, during the fermentation process (31). ‘Frontenac' creates a red wine, and to achieve high quality, the grower must retain a deep red color. Given our results from our 2021 trial, it demonstrates that *P. japonica* feeding results in decreased tannins present, which in turn will impact the wines retention of anthocyanins. To correct this during the wine making process, growers will need to add additional tannins to ensure their red wine retains the proper anthocyanins. Table 1 further demonstrates this phenomenon when we observe the anthocyanin measurements across treatments.

Results from these trials consistently demonstrate that as beetle density (>50 beetles per m-row) and defoliation (>30%) increase, there is a significant and negative impact on the quality of the fruit. Boucher et al. (32), concluded that just after a 20% leaf area loss, net photosynthesis began to decline quickly. This observation agrees with our results that when >30% defoliation by *P. japonica* is observed, negative impacts on the quality of the fruit begin to develop.

Thus far, only limited research has been conducted on this topic, but our studies agree with past literature from Boucher and Pfeiffer (10) where similar trials demonstrated that with high enough defoliation, fruit quality parameters such as SSC are negatively impacted in the wine grape variety ‘Seyval Blanc.' Another study, published on the impact of *P. japonica* to young vines in Michigan, demonstrated little impact to the vine's vegetative growth (11). However, in the Michigan study, beetles were only exposed to the vines for 2 weeks starting at véraison and infested at much lower levels with the highest being 40 beetles per cage. Also, because the focus of these trials was on young vines that were not yet fruiting, it is difficult to compare our studies directly, as our vines were ~7 years old and producing fruit. Furthermore, our trials had beetles feeding on the vine after the berries were at the pea-size stage and until harvest occurred creating a much longer window for beetle feeding to impact the crop. Finally, Hammons et al. (13) indicates how varieties may differ in both susceptibility and response to *P. japonica* foliar feeding. Our study focused exclusively on ‘Frontenac' whereas Hammons et al. (12) compared yield and quality parameters of 5 different varieties. Results indicated yield impacts varied between the different varieties, meaning some varieties exhibited a decrease in cluster yield such as ‘Cabernet Franc' and ‘Norton,' while ‘Concord' did not. Furthermore, none of the tested varities exhibited any significant impact to fruit quality. Results from this study emphasize the importance of continuing trials on other wine grapes to understand how different varieties respond to *P. japonica* foliar feeding.

In conclusion, results indicate that *P. japonica* feeding has a negative impact on the development of ‘Frontenac' berries after ~30–35% defoliation, and >50 *P. japonica* per m-row. These data support the use of a tentative action threshold of 25–30% defoliation or >25 *P. japonica* per m-row would warrant management action to minimize negative impacts to the quality parameters of the crop. Trials were conducted in an area of Minnesota where *P. japonica* has not yet exhibited high natural densities, which explains the low densities recorded in open plots. However, observations in other areas of Minnesota, where *P. japonica* is better established, often yield >25 beetles per-m row during peak beetle activity in mid to late summer (personal observation). This study is specific to ‘Frontenac' and with the limited number of studies published on wine grapes (10–13), there is a critical need for additional research in other varieties. In areas where *P. japonica* continues to invade new regions, including Europe (33), it will be important to continue these studies on other varieties in different climates and potentially evaluate long term impacts to vines and berry quality after successive years of beetle injury. Moreover, in areas where *P. japonica* is now established such as Minnesota, climate change projections during the twenty first century of additional increases of 4°C increase summer months, and 6°C increase during winters (34) portend additional abiotic benefits to *P. japonica* population growth, overwintering success, and potential range expansion (33). Expanding our knowledge of the impact *P. japonica* has on high value crops such as wine grapes, will benefit growers and other stakeholders in finding sustainable IPM solutions for managing the pest now, and into the future as the pest is likely to invade new areas.

## Data Availability Statement

The raw data supporting the conclusions of this article will be made available by the authors, upon request.

## Author Contributions

DE, EB, and WH: conceptualization. WH, EB, and MC: resources, project administration, and funding acquisition. DE, EB, and MC: methodology. DE: investigation and formal analysis. DE: writing original draft, and preparation. DE, EB, MC, and WH: writing. All authors provided a final review, and agreed to the published version of the manuscript.

## Funding

The research was funded by the Rapid Agricultural Response Fund, Award 0081527, via the Minnesota Agricultural Experiment Station, University of Minnesota. The project was also supported by the McLaughlin Gormley King (MGK) Fellowship, and a University of Minnesota Doctoral Dissertation Fellowship.

## Conflict of Interest

WH is a topic editor for the *P. japonica* RT, an independent Assoc. Editor was selected to manage the review process. The remaining authors confirm that the research was conducted in the absence of any commercial or financial relationships that could be construed as a potential conflict of interest.

## Publisher's Note

All claims expressed in this article are solely those of the authors and do not necessarily represent those of their affiliated organizations, or those of the publisher, the editors and the reviewers. Any product that may be evaluated in this article, or claim that may be made by its manufacturer, is not guaranteed or endorsed by the publisher.

## References

[1] Fleming WE. Integrating Control of the Japanese Beetle - A Historical Review. Technical Bulletin No. 1545. Washington, DC: USDA (1976).

[2] Hahn J Weisenhorn J Bugeja S. Japanese Beetles in Yards Gardens. University of Minnesota Extension, St. Paul, MN (2020). Available online at: https://extension.umn.edu/yard-and-garden-insects/japanese-beetles (accessed December 23, 2021).

[3] Potter DA Held DW. Biology and management of the Japanese beetle. Annu Rev Entomol. (2002) 47:175–205. 10.1146/annurev.ento.47.091201.145153 11729073

[4] Shanovich HN Ashley DN Koch RL Hodgson EW. Biology and management of Japanese beetle (Coleoptera: Scarabaeidae) in corn and soybean. J Integ Pest Manage. (2019) 10:1–14. 10.1093/jipm/pmz009

[5] Althoff EM Rice KB. Japanese beetle (Coleoptera: Scarabaeidae) invasion of North America: history, ecology, and management. J Integ Pest Manage. (2022) 13:1–11. 10.1093/jipm/pmab043

[6] USDA: Animal and Plant Health Inspection Service. Japanese Beetle Distribution in the U.S (2022). Available online at: https://www.aphis.usda.gov/aphis/ourfocus/planthealth/plant-pest-and-disease-programs/pests-and-diseases/japanese-beetle/japanese-beetle. (accessed February 22, 2022).

[7] Potter DA. Destructive Turfgrass Insects: Biology, Diagnosis, and Control. Chelsea, MI: Ann Arbor Press (1998). p. 368.

[8] Fleming WE. Biology of the Japanese Beetle. Technical Bulletin No. 1449. Washington, DC: USDA (1972).

[9] Vittum PJ Villani MG Tashiro H. Turfgrass Insects of the United States and Canada. 2nd ed. Ithaca, NY: Cornell University Press (1999).

[10] Boucher TJ Pfeiffer DG. Influence of Japanese beetle (Coleoptera: Scarabaeidae) foliar feeding on ‘Seyval Blanc' grapevines in Virginia. J Econ Entomol. (1989) 82:220–5. 10.1093/jee/82.1.220

[11] Mercader RJ Isaacs R. Damage potential of Rose Chafer and Japanese beetle (Coleoptera: Scarabaeidae) in Michigan vineyards. Great Lakes Entomol. (2003) 36:1–13.

[12] Hammons DL Kurtural SK Potter DA. Japanese beetle defoliation reduces primary bud cold-hardiness during vineyard establishment. Am J Enol Vitic. (2010) 61:130–4.

[13] Hammons DL Kurtural SK Potter DA. Impact of insecticide-manipulated defoliation by Japanese beetle (*Popillia japonica*) on grapevines for vineyard establishment through production. Pest Manag Sci. (2010) 66:565–71. 10.1002/ps.1908 20101596

[14] Pfeiffer DG. Japanese beetle and other Coleoptera Feeding on Grapevines in Eastern North America. In: Bostanian NJ, Vincent C, Isaacs R, editors. Arthropod Management in Vineyards: Pests, Approaches, and Future Directions. Dordrecht: Springer (2012). p. 403–29.

[15] Ollat N Carde J Gaudillére J Barrieu F Diakou-Verden P Moing A. Grape berry development: a review. J Int Sci Vigne du Vin. (2002) 36:109–31. 10.20870/oeno-one.2002.36.3.970

[16] Ebbenga DN Wold-Burkness SJ Burkness EC Hutchison WD. Japanese Beetle: An Emerging Pest of Fruit Crops. Pest Profile, FruitEdge, University of Minnesota, St. Paul (2021). Available online at: http://www.fruitedge.umn.edu/japanese-beetle/japanese-beetle-emerging-pest-fruit-crops (accessed January 31, 2022).

[17] Amerine MA Roessler EB. Field testing grape maturity. Hilgardia. (1958) 28:93–114. 10.3733/hilg.v28n04p093

[18] Berg HW Ough CS. The relation of balling to wine quality. Am J Enol Viticult. (1977) 28:235–8.

[19] Du Plessis CS. Optimum maturity and quality parameters in grapes: a review. S Afr J. Enol Viti. (1984) 5:35–42. 10.21548/5-1-2367

[20] Tuck B Gartner W Appiah G. Economic Contribution of Vineyards Wineries of the North, 2015. University of Minnesota Extension, St. Paul, MN (2017). Available online at: https://conservancy.umn.edu/bitstream/handle/11299/197808/2015-economic-contribution-wineries-and-grapes.pdf?sequence=1 (accessed December 23, 2021).

[21] Hemstad P. Grapevine breeding in the Midwest. In: Reynolds AG, editors. Grapevine Breeding Programs for the Wine Industry. Cambridge: Woodhead (2015). p. 411–25.

[22] Meier U. Grapevine. Growth Stages of Mono- and Dicotyledonous Plants. BBCH Monograph. Federal Biological Research Centre for Agriculture and Forestry, Berlin (2001). p. 204.

[23] Rogers MA Burkness EC Hutchison WD. Evaluation of high tunnels for management of Drosophila suzukii in fall-bearing red raspberries: potential for reducing insecticide use. J Pest Sci. (2016) 89:815–21. 10.1007/s10340-016-0731-1

[24] Ebbenga DN Burkness EC Hutchison WD. Evaluation of exclusion netting for Spotted- wing drosophila (Diptera: Drosophilidae) management in Minnesota wine grapes. J Econ Entomol. (2019) 112:2287–94. 10.1093/jee/toz143 31143945 PMC6756779

[25] Ebbenga DN Burkness EC Clark MD Hutchison WD. Risk of spotted-wing drosophila injury and associated increases in volatile acidity in Minnesota wine grapes. Am J Enol Vitic. (2021) 72:106–12. 10.5344/ajev.2020.20008

[26] Getman-Pickering ZL Campbell A Aflitto N Grele A Davis JK Ugine TA. LeafByte: a mobile application that measures leaf area and herbivory quickly and accurately. Methods Ecol Evol. (2020) 11:215–21. 10.1111/2041-210X.13340

[27] Coombe BG. Research on development and ripening of the grape berry. Am J Enol. Vitic. (1992) 43:101–10.

[28] R Core Team. R: A Language Environment for Statistical Computing. Vienna (2017). Available online at: http://www.r-project.org/

[29] Mendiburu FD. Agricolae: Statistical Procedures for Agricultural Research. R package version (2015). p. 2–3. Available online at: http://CRAN.R-project.org/package=agricolae (accessed February 20, 2021).

[30] Clark MD. Cold Hardy Wine Grapes: Frontenac. University of Minnesota Extension, St. Paul, MN (2020). Available online at: https://enology.umn.edu/sites/enology.umn.edu/files/2021-10/409_frontenacfactsheet_fin.pdf (accessed December 15, 2021).

[31] Fournand D Vicens A Sidhoum L Souquet J Moutounet M Cheynier V. Accumulation and extractability of grape skin tannins and anthocyanins at different advanced physiological stages. J Agric Food Chem. (2006) 54:7331–8. 10.1021/jf061467h 16968102

[32] Boucher TJ Pfeiffer DG Barden JA Williams JM. Effects of simulated insect injury on net photosynthesis of potted grapevines. Hortscience. (1987) 22:927–8.

[33] Kistner-Thomas EJ. The potential global distribution and voltinism of the Japanese beetle (Coleoptera: Scarabaeidae) under current and future climates. J Insect Sci. (2019) 9:1–13. 10.1093/jisesa/iez023 30900722 PMC6429693

[34] Liess S Twine TE Snyder PK Hutchison WD Konar-Steenberg G Keeler BL. High-resolution climate projections over Minnesota for the 21st century. Earth Space Sci. (2022) 9:e2021EA.001893. 10.1029/2021EA001893

